# Pretreatment Inflammatory Indexes as Prognostic Predictors for Survival in Colorectal Cancer Patients Receiving Neoadjuvant Chemoradiotherapy

**DOI:** 10.1038/s41598-018-21093-7

**Published:** 2018-02-14

**Authors:** Jing Yang, Hui Xu, Xinli Guo, Jing Zhang, Xiaoyang Ye, Yanping Yang, Xuelei Ma

**Affiliations:** 1 State Key Laboratory of Biotherapy and Cancer Center, West China Hospital, Sichuan University, and Collaborative Innovation Center for Biotherapy, Chengdu, China; 2 West China School of Medicine, West China Hospital, Sichuan University, Chengdu, 610041 PR China

## Abstract

This study was to evaluate the prognostic value of pretreatment inflammatory indexes including neutrophil-to-lymphocyte ratio (NLR), platelet-to-lymphocyte ratio (PLR), lymphocyte-to-monocyte ratio (LMR), and systemic immune-inflammation index (SII) in colorectal cancer (CRC) patients receiving neoadjuvant chemoradiotherapy (CRT). We enrolled 98 eligible CRC patients and divided them into high or low NLR, PLR, LMR, and SII groups according to their median index value, respectively. Univariate and multivariate analysis were performed to identify the potential predictors of progression-free survival (PFS) and overall survival (OS). In the univariate analysis, ECOG performance status, distant metastasis, NLR, PLR, LMR, and SII were found to be significantly associated with PFS and OS. In the multivariate analysis, ECOG performance status, distant metastasis, and NLR were identified to be independent predictors of PFS (HR 2.487, p = 0.012; HR 2.422, p = 0.042; HR 2.243, p = 0.034, respectively), and OS (HR 2.237, p = 0.018; HR 2.757, p = 0.020; HR 2.336, p = 0.017, respectively). The results of our study revealed that ECOG performance status, distant metastasis and NLR were independent prognostic factors of PFS and OS in CRC patients receiving neoadjuvant CRT.

## Introduction

Colorectal cancer (CRC) is the third most commonly diagnosed cancer and the fourth leading cause of cancer-related death worldwide^1^. Great progress has been made in surgery, radiotherapy, and drug therapy for CRC treatment in the past few years^2^, which significantly decrease local recurrence rates and greatly improve survival rates of CRC patients^3–5^. Several randomized controlled trials have reported that neoadjuvant radiotherapy is more effective and less toxic than adjuvant radiotherapy among CRC patients^6–8^. In addition, patients receiving preoperative chemoradiotherapy (CRT) have been reported to have better local control than those receiving preoperative radiotherapy alone^9–11^.

Previous investigations have indicated that inflammatory indexes such as neutrophil-to-lymphocyte ratio (NLR), platelet-to-lymphocyte ratio (PLR), lymphocyte-to-monocyte ratio (LMR) and systemic immune-inflammation index (SII) play important roles in the prediction of survival in various types of malignant tumor, including CRC, pancreatic cancer, ovarian cancer, breast cancer, gastric cancer and esophageal cancer^12–18^. Although pretreatment inflammatory indexes have been demonstrated to be associated with survival in CRC patients undergoing surgical resection and chemotherapy, the prognostic value of those indexes has not been well evaluated in CRC patients receiving neoadjuvant CRT^19,20^.

The aim of this study was to investigate pretreatment parameters including NLR, PLR, LMR and SII for their ability to predict survival of CRC patients receiving neoadjuvant CRT.

## Results

### Baseline characteristics

A total of 7207 patients with CRC were identified in the database and 98 of them were enrolled in the present study. We divided patients into high and low indexes groups on the basis of the median index value of NLR (2.22), PLR (114.15), LMR (4.27), and SII (437.72), respectively. NLR ≥ 2.22, PLR ≥ 114.15, LMR ≥ 4.27, and SII ≥ 437.72 were considered as elevated groups.

The baseline characteristics of patients are shown in Table 1. The median age of patients was 53 (range 26–83) years. There were 59 (60.2%) males and 39 (39.8%) females. Eighty-one patients (82.7%) of them had an ECOG performance status of 0, whereas 17 (17.3%) of them had an ECOG performance status of 1–2. Of the entire patients, 67 (68.4%) had tumor located in left and right colon, whereas 31(31.6%) in rectum. Among the 98 patients, 40 (40.8%) had distant metastasis. All the 98 patients received neoadjuvant chemotherapy, 32 (32.6%) received FOLFOX, 48 (49.0%) received XELOX and 18 (18.4%) received FOLFIRI. Regarding histologic grade, 10 (18.5%) cases were low-grade (G1) CRC, whereas 44 (81.5%) patients were intermediate-grade (G2) or high-grade (G3) CRC.

**Table 1:** Baseline characteristics of the study population (n = 98).

	**NLR**		***P***	**PLR**		***p***	**LMR**		***p***	SII		***p***
	**<2.22**	≥**2.22**		**<114.15**	≥**114.15**		**<4.27**	≥**4.27**		**<437.72**	≥**437.72**	
	***n*** **(%)**	***n*** **(%)**		***n*** **(%)**	***n*** **(%)**		**n(%)**	**n(%)**		**n(%)**	**n(%)**	
**Median age, years (range)**	53(26–83)	53(26–78)	0.872	53(26–83)	52(26–78)	0.741	57(28–77)	50(26–83)	0.084	52(26–83)	54(28–78)	0.367
**Gender**												
Male	25(51.0)	34(69.4)	0.063	27(55.1)	32(65.3)	0.302	29(59.2)	30(61.2)	0.836	26(53.1)	33(67.3)	0.149
Female	24(49.0)	15(30.6)		22(44.9)	17(34.7)		20(40.8)	19(38.8)		23(46.9)	16(32.7)	
**ECOG performance status**												
0	41(83.7)	40(81.6)	0.790	39(79.6)	42(85.7)	0.424	39(79.6)	42(85.7)	0.424	43(87.8)	38(77.6)	0.182
1–2	8(16.3)	9(18.4)		10(20.4)	7(14.3)		10(20.4)	7(14.3)		6(12.2)	11(22.4)	
**Tumor location**												
Left colon	17(34.7)	16(32.7)	0.914	15(30.6)	18(36.7)	0.810	16(32.7)	17(34.7)	0.969	15(30.6)	18(36.7)	0.139
Right colon	16(32.7)	18(36.7)		18(36.7)	16(32.7)		17(34.7)	17(34.7)		14(28.6)	20(40.8)	
Rectum	16(32.7)	15(30.6)		16(32.7)	15(30.6)		16(32.7)	15(30.6)		20(40.8)	11(22.4)	
**Clinical T stage** ^a^												
T1–2	5(13.5)	4(9.3)	0.726	3(8.1)	6(14.0)	0.494	4(9.5)	5(13.2)	0.729	5(12.8)	4(9.8)	0.734
T3–4	32(86.5)	39(90.7)		34(91.9)	37(86.0)		38(90.5)	33(86.8)		34(87.2)	37(90.2)	
**Lymphnode metastasis** ^a^												
No	9(24.3)	8(18.6)	0.533	9(24.3)	8(18.6)	0.533	7(16.7)	10(26.3)	0.292	8(21.6)	9(20.9)	0.940
Yes	28(75.7)	35(81.4)		28(75.7)	35(81.4)		35(83.3)	28(73.7)		29(78.4)	34(79.1)	
**Distant metastasis**												
No	39(79.6)	19(38.8)	<0.001	38(77.6)	20(40.8)	<0.001	16(32.7)	42(85.7)	<0.001	40(81.6)	18(36.7)	<0.001
Yes	10(20.4)	30(61.2)		11(22.4)	29(59.2)		33(67.3)	7(14.3)		9(18.4)	31(63.3)	
**Neoadjuvant chemotherapy regimen**												
FOLFOX	17(34.7)	15(30.6)	0.578	15(30.6)	17(34.7)	0.841	11(22.4)	21(42.9)	0.042	19(38.8)	13(26.5)	0.262
XELOX	25(51.0)	23(46.9)		24(49.0)	24(49.0)		30(61.2)	18(36.7)		20(40.8)	28(57.1)	
FOLFIRI	7(14.3)	11(22.4)		10(20.4)	8(16.3)		8(16.3)	10(20.4)		10(20.4)	8(16.3)	
**Pathological differentiation** ^b^												
Low grade	4(16.7)	6(20.0)	1.000	5(20.0)	5(17.2)	1.000	3(10.0)	7(29.2)	0.089	4(16.0)	6(20.7)	0.736
Intermediate to high grade	20(83.3)	24(80.0)		20(80.0)	24(82.8)		27(90.0)	17(70.8)		21(84.0)	23(79.3)	
**LDH**												
≤220	47(95.9)	46(93.9)	1.000	46(93.9)	47(95.9)	0.382	44(89.8)	49(100.0)	0.056	48(98.0)	45(91.8)	1.000
>220	2(4.1)	3(6.1)		3(6.1)	2(4.1)		5(10.2)	0(0.0)		1(2.0)	4(8.2)	
**CA 19-9** ^c^												
≤22	27(61.4)	33(71.7)	0.297	26(61.9)	34(70.8)	0.370	28(62.2)	32(71.1)	0.371	32(72.7)	28(60.9)	0.233
>22	17(38.6)	13(28.3)		16(38.1)	14(29.2)		45(37.8)	45(28.9)		12(27.3)	18(39.1)	
**CA-125** ^d^												
≤35	35(97.2)	38(97.4)	1.000	35(100.0)	38(95.0)	0.495	33(97.1)	40(97.6)	1.000	36(97.3)	37(97.4)	1.000
>35	1(2.8)	1(2.6)		0(0.0)	2(5.0)		1(2.9)	1(2.4)		1(2.7)	1(2.6)	

^a^80 were available, ^b^54 were available, ^c^90 were available, ^d^75 were available.

NLR neutrophil-lymphocyte ratio, PLR platelet-lymphocyte ratio, LMR lymphocyte-monocyte ratio, SII systemic immune-inflammation index, LDH lactic dehydrogenase, CA 19-9 carbohydrate antigen 19-9, CA-125 carbohydrate antigen-125.

Forty-eight of the 98 patients were alive after a median follow-up of 37.0 months (range 16.2–93.3 months). The overall median progression-free survival (PFS) was 16.4 months (95% confidence interval [CI] 16.6–22.3), and the median overall survival (OS) was 25.8 months (95% CI 26.7–31.8). We investigated the associations between age, gender, ECOG performance status, tumor location, clinical T stage, lymph node metastasis, distant metastasis, neoadjuvant chemotherapy regimen, pathological differentiation, NLR, PLR, LMR, SII, lactic dehydrogenase (LDH), carbohydrate antigen 19-9 (CA 19-9) and survival by performing Cox proportional hazard regression analysis.

### Univariate analysis and multivariate analysis

**Figure 1:**
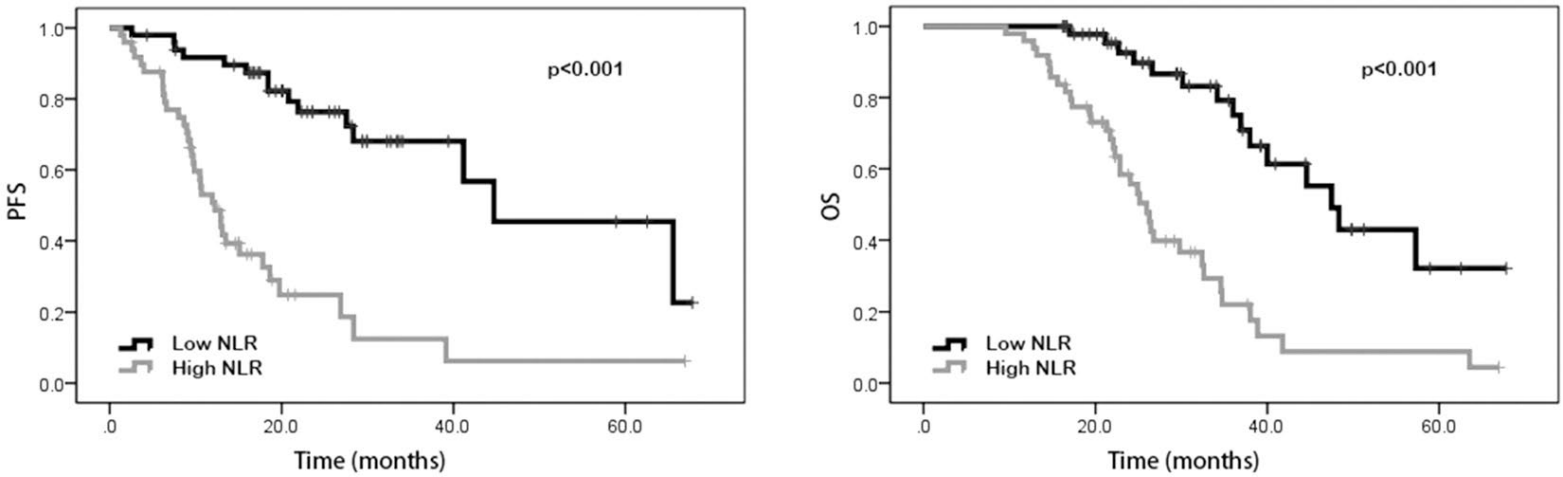
Kaplan-Meier curves of progression-free survival (PFS) and overall survival (OS) according to neutrophil-to-lymphocyte ratio (NLR) values.

**Figure 2:**
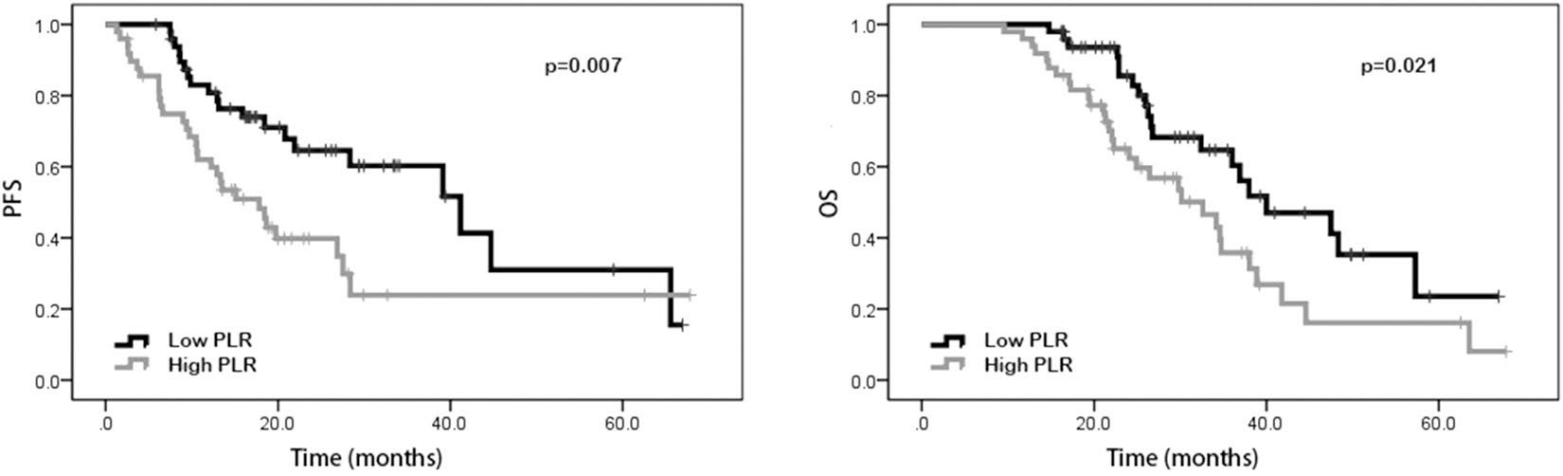
Kaplan-Meier curves of progression-free survival (PFS) and overall survival (OS) according to platelet-to- lymphocyte ratio (PLR) values.

**Figure 3:**
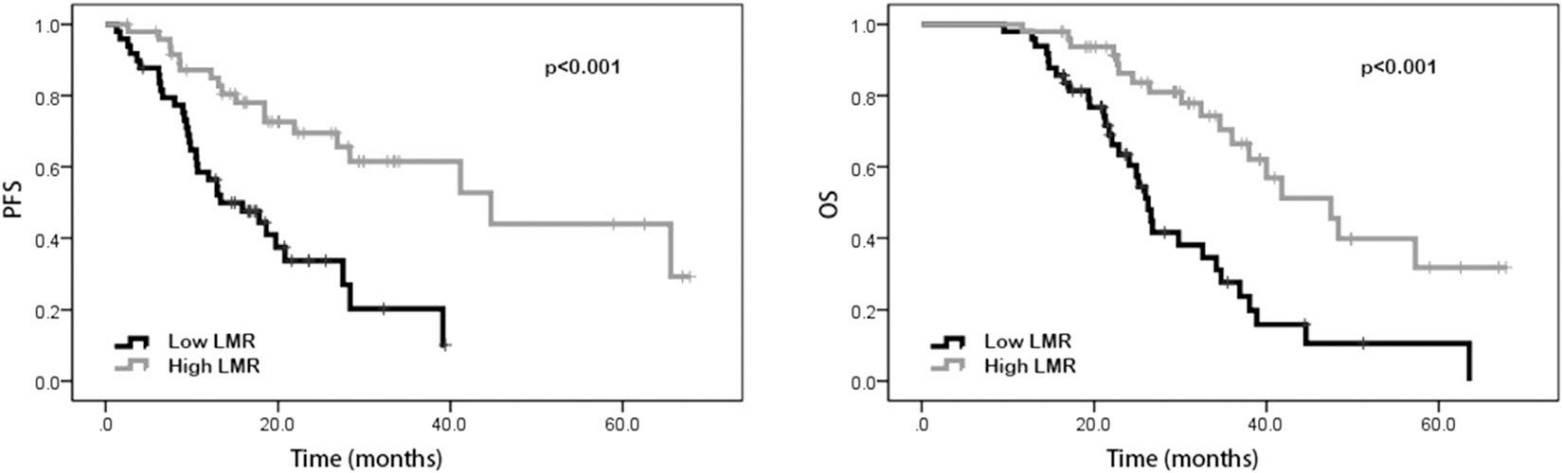
Kaplan-Meier curves of progression-free survival (PFS) and overall survival (OS) according to lymphocyte-to-monocyte-ratio (LMR) values.

**Figure 4:**
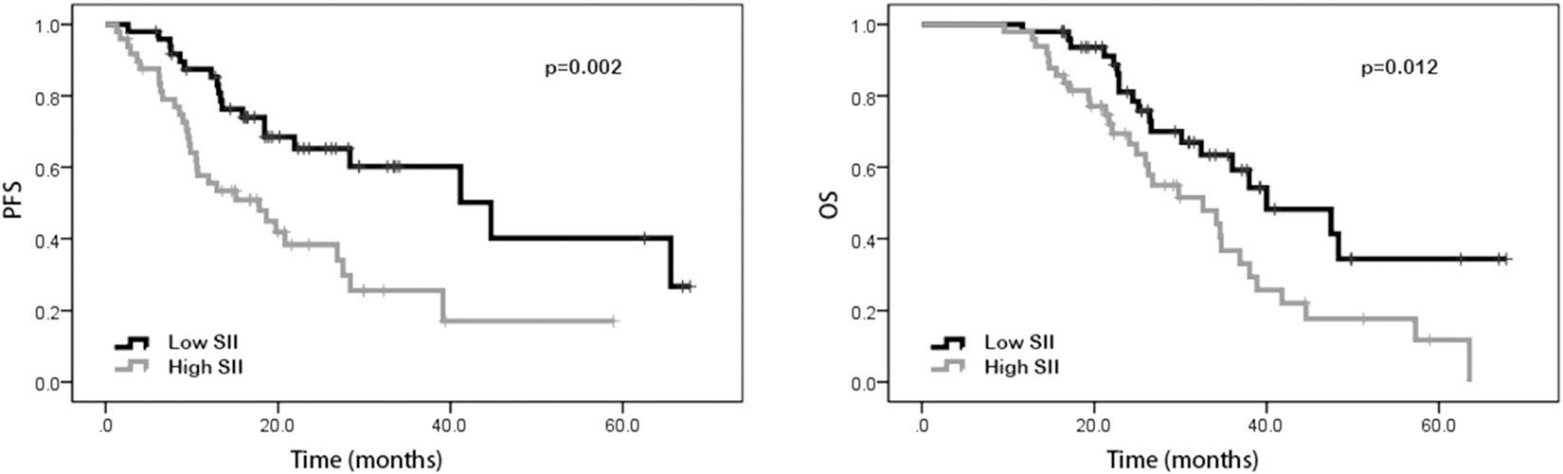
Kaplan-Meier curves of progression-free survival (PFS) and overall survival (OS) according to systemic immune-inflammation index (SII) values.

**Figure 5:**
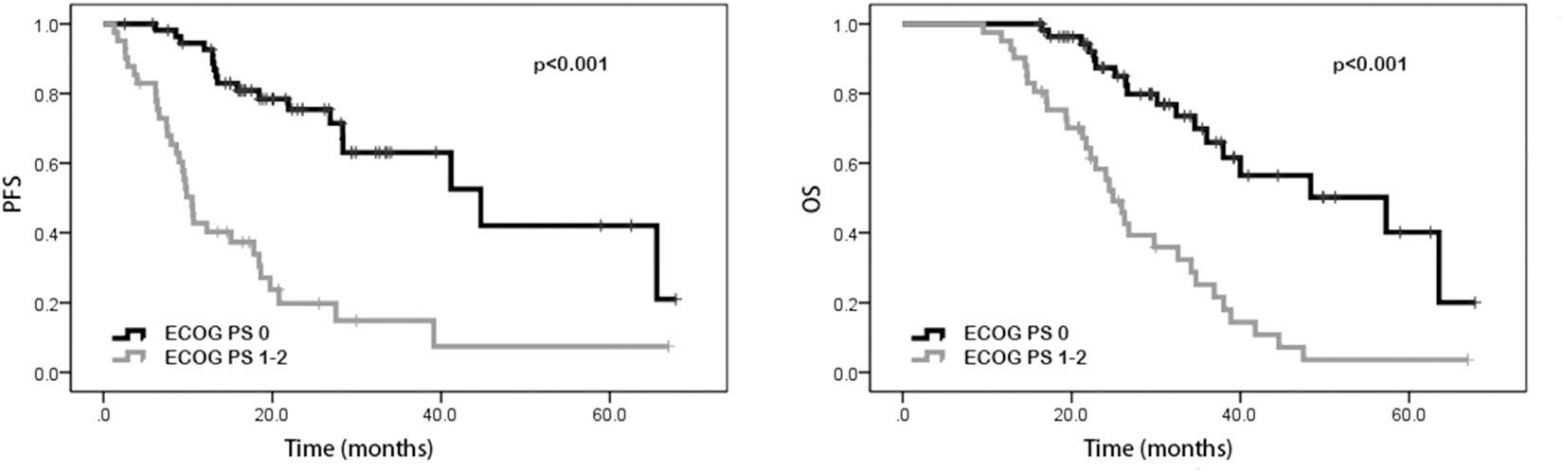
Kaplan-Meier curves of progression-free survival (PFS) and overall survival (OS) according to ECOG performance status (ECOG PS).

**Figure 6:**
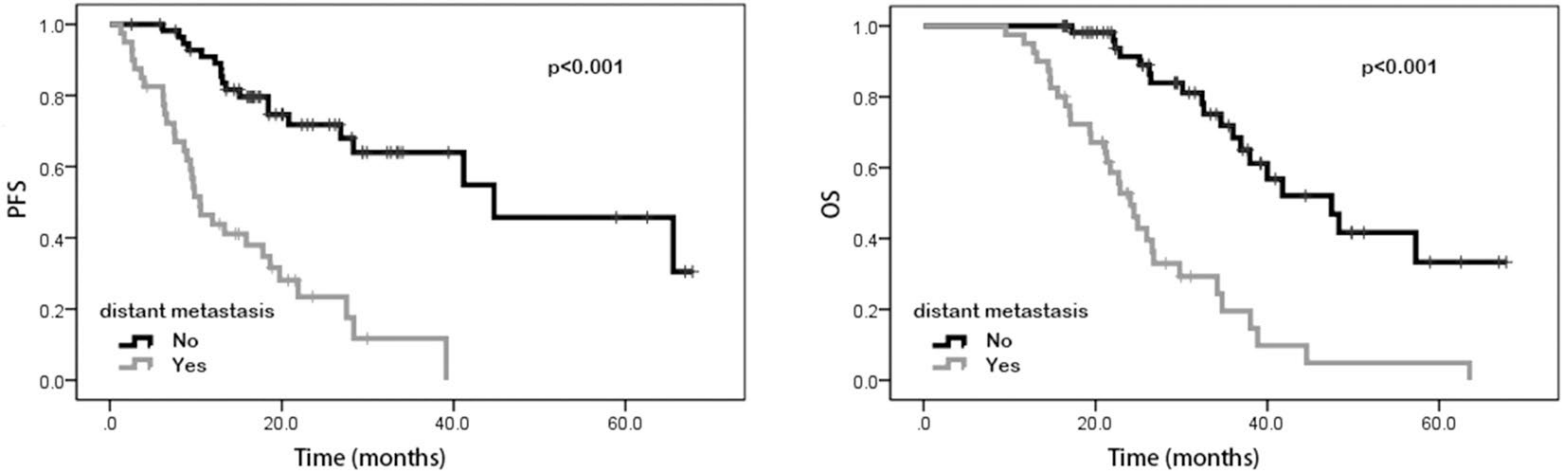
Kaplan-Meier curves of progression-free survival (PFS) and overall survival (OS) according to distant metastasis.

In the univariate analysis, our results suggested that patients with performance status of 0 possessed better PFS (20.0 vs. 9.8 months, p < 0.001) and OS (29.4 vs. 22.3 months, p < 0.001) than those with performance status of 1–2. Favorable PFS (18.9 vs. 10.2 months, p < 0.001) and OS (30.5 vs. 22.2 months, p < 0.001) were also found in patients without distant metastasis. Patients with high NLR and PLR were shown to have poorer PFS (23.0 vs. 10.7 months, p < 0.001 and 18.4 vs. 13.5 months, p = 0.009, respectively) and OS (30.1 vs. 22.9 months, p < 0.001 and 26.6 vs. 23.6 months, p = 0.023, respectively) than patients with low NLR and PLR. Patients with high LMR were shown to have better PFS (12.9 vs. 20.0 months, p < 0.001) and OS (22.1 vs. 30.9 months, p < 0.001) than those with low LMR. Compared to those with low SII, patients with high SII were shown to have worse PFS (18.5 vs. 13.5 months, p = 0.002) and OS (26.6 vs. 23.6 months, p = 0.013). However, age, gender, tumor location, pathological differentiation, clinical T stage, lymph node metastasis, neoadjuvant chemotherapy regimen, LDH, and CA 19-9 were not shown to be associated with PFS and OS (Tables 2 and 3). In addition, Kaplan–Meier curve also showed that ECOG performance status, distant metastasis, NLR, PLR, LMR and SII were significantly associated with PFS and OS (Figs 1, 2, 3, 4, 5, and 6).

**Table 2:** Univariate and multivariate analysis of PFS.

Variable	Parameter	Median PFS	95% CI	Univariate analysis		Multivariate analysis	
				HR (95% CI)	*p* value	HR (95% CI)	*p* value
Age	<54	16.7	16.6–25.9	1.000	0.543	—	—
	≥54	15.9	14.2–20.0	1.195 (0.673–2.121)		—	
Gender	Male	15.0	14.5–21.7	1.000	0.762	—	—
	Female	18.4	16.6–26.3	0.917 (0.522–1.610)		—	
ECOG performance status	0	20.0	19.9–27.5	1.000	<0.001	1.000	0.012
	1–2	9.8	9.7–17.3	4.645 (2.579–8.365)		2.487 (1.221–5.063)	
Tumor location	Left colon	18.6	16.1–26.3	1.000	0.620	—	—
	Right colon	14.1	12.3–22.7	0.717 (0.366–1.402)		—	
	Rectum	16.5	14.6–24.6	0.832 (0.422–1.642)		—	
Clinical T stage	T2	7.6	4.8–19.8	1.000	0.860	—	—
	T3	14.9	14.4–24.4	1.352 (0.458–3.989)		—	
	T4	16.4	14.9–25.8	1.068 (0.569–2.005)		—	
Lymph node metastasis	No	13.5	11.2–22.9	1.000	0.824	—	—
	Yes	15.9	15.6–23.5	0.919 (0.438–1.929)		—	
Distant metastasis	No	18.9	19.9–28.1	1.000	<0.001	1.000	0.042
	Yes	10.2	10.0–15.6	0.445 (0.321–0.618)		2.422 (1.031–5.692)	
Neoadjuvant chemotherapy regimen	FOLFOX	17.2	16.6–29.9	1.000	0.914	—	—
	XELOX	16.6	15.1–20.7	1.194 (0.503–2.834)		—	
	FOLFIRI	13.2	9.1–24.4	1.093 (0.466–2.564)		—	
Pathological differentiation	Low grade	12.0	6.5–31.1	1.000	0.837	—	—
	Intermediate grade	14.5	13.8–23.2	1.445 (0.359—5.817)		—	
	High grade	16.7	8.4–23.2	1.436 (0.430–4.794)		—	
NLR	<2.22	23.0	21.5–30.0	1.000	<0.001	1.000	0.034
	≥2.22	10.7	10.1–16.2	5.101 (2.719–9.572)		2.243 (1.061–4.738)	
PLR	<114.15	18.4	18.7–27.1	1.000	0.009	1.000	0.277
	≥114.15	13.5	12.2–19.8	2.151 (1.215–3.808)		1.464 (0.737–2.910)	
LMR	<4.27	12.9	12.0–17.1	1.000	<0.001	1.000	0.489
	≥4.27	20.0	19.5–29.1	0.308 (0.165–0.575)		0.729 (0.297–1.786)	
SII	<437.72	18.5	18.6–27.7	1.000	0.002	1.000	0.244
	≥437.72	13.5	12.7–19.0	2.498 (1.387–4.499)		0.569 (0.220–1.470)	
LDH	<220	16.4	16.4–22.4	1.000	0.722	—	—
	≥220	16.7	3.0–36.0	0.773 (0.187–3.190)		—	
CA19–9	<22	16.2	15.6–23.2	1.000	0.394	—	—
	≥22	15.9	13.7–22.5	1.295 (0.715–2.347)		—	

NLR neutrophil-to-lymphocyte ratio, PLR platelet-to-lymphocyte ratio, LMR lymphocyte-to-monocyte ratio, SII systemic immune-inflammation index, LDH lactic dehydrogenase, CA 19-9 carbohydrate antigen 19-9.

**Table 3:** Univariate and multivariate analysis of OS.

Variable	Parameter	Median OS	95% CI	Univariate analysis		Multivariate analysis	
				HR (95% CI)	*p* value	HR (95% CI)	*p* value
Age	<54	26.4	27.1–35.0	1.000	0.259	—	—
	≥54	25.5	23.8–29.8	1.394 (0.783–2.484)		—	
Gender	Male	28.2	25.9–32.4	1.000	0.663	—	—
	Female	25.5	24.8–33.7	1.137 (0.639–2.021)		—	
ECOG performance status	0	29.4	28.5–35.5	1.000	<0.001	1.000	0.018
	1–2	22.3	21.7–29.0	3.907 (2.177–7.012)		2.237 (1.146–4.367)	
Tumor location	Left colon	26.8	25.7–35.6	1.000	0.657	—	—
	Right colon	23.7	23.0–31.5	0.733 (0.376–1.429)		—	
	Rectum	26.2	25.1–34.4	0.847 (0.429–1.617)		—	
Clinical T stage	T2	30.1	22.5–35.8	1.000	0.551	—	—
	T3	23.2	23.3–30.8	0.940 (0.314–2.813)		—	
	T4	26.3	25.4–36.6	1.392 (0.726–2.670)		—	
Lymph node metastasis	No	30.1	24.2–38.0	1.000	0.644	—	—
	Yes	24.9	25.1–32.1	1.191 (0.568–2.495)		—	
Distant metastasis	No	30.5	29.3–36.3	1.000	<0.001	1.000	0.020
	Yes	22.2	20.7–27.2	0.464 (0.340–0.635)		2.757 (1.171–6.492)	
Neoadjuvant chemotherapy regimen	FOLFOX	33.0	29.6–39.8	1.000	0.575	—	—
	XELOX	24.5	23.6–29.0	1.041 (0.432–2.510)		—	
	FOLFIRI	22.6	19.9–35.1	1.402 (0.583–3.370)		—	
Pathological differentiation	Low grade	25.6	20.1–39.2	1.000	0.985	—	—
	Intermediate grade	26.4	26.6–35.9	1.099 (0.274–4.413)		—	
	High grade	22.3	15.3–33.8	1.017 (0.303–3.408)		—	
NLR	<2.22	30.1	29.2–36.8	1.000	<0.001	1.000	0.017
	≥2.22	22.9	22.2–28.6	4.204 (2.260–7.820)		2.336 (1.165–4.687)	
PLR	<114.15	26.6	27.3–34.6	1.000	0.023	1.000	0.954
	≥114.15	23.6	23.8–31.1	1.934 (1.093–3.419)		0.978 (0.466–2.054)	
LMR	<4.27	22.1	22.1–28.2	1.000	<0.001	1.000	0.125
	≥4.27	30.9	29.3–37.2	0.315 (0.175–0.567)		0.446 (0.159–1.252)	
SII	<437.72	26.6	27.3–34.8	1.000	0.013	1.000	0.266
	≥437.72	23.6	23.8–31.0	2.059 (1.161–3.650)		0.572 (0.213–1.531)	
LDH	<220	26.0	26.9–32.2	1.000	0.668	—	—
	≥220	19.5	12.1–33.7	1.366 (0.328–5.684)		—	
CA19-9	<22	25.5	26.1–33.3	1.000	0.333	—	—
	≥22	26.4	25.0–33.7	1.344 (0.739–2.445)		—	

NLR neutrophil-to-lymphocyte ratio, PLR platelet-to-lymphocyte ratio, LMR lymphocyte-to-monocyte ratio, SII systemic immune-inflammation index, LDH lactic dehydrogenase, CA 19-9 carbohydrate antigen 19-9.

Subsequently, multivariate analysis was performed to assess the independent predictors for survival. Our results revealed that performance status of 1–2, distant metastasis and high NLR were independent predictors of poor survival. Patients with performance status of 1–2 were shown to have poorer PFS (hazard ratio [HR] 2.487, 95% CI 1.221–5.063, p = 0.012) and OS (HR 2.237, 95% CI 1.146–4.367, p = 0.018) than those with performance status of 0. Patients with distant metastasis were shown to have remarkably worse PFS (HR 2.422, 95% CI 1.031–5.692, p = 0.042) and OS (HR 2.757, 95% CI 1.171–6.492, p = 0.020) than patients without distant metastasis. Patients with elevated NLR were shown to have poor PFS (HR 2.243, 95% CI 1.061–4.738, p = 0.034) and OS (HR 2.336, 95% CI 1.165–4.687, p = 0.017) than patients with low NLR (Tables 2 and 3).

## Discussion

Inflammatory indexes were identified as important prognostic indicators in patients with CRC, nevertheless, the prognostic value of those indexes among CRC patients receiving neoadjuvant CRT has not been fully established so far^19,20^. To the best of our knowledge, in this research we firstly reported the prognostic significance of inflammatory indexes in CRC patients receiving neoadjuvant CRT. Our results revealed that ECOG performance status, distant metastasis, NLR, PLR, LMR and SII were strongly associated with PFS and OS. Other factors including sex, age, tumor localization, clinical T stage, lymph node metastasis, neoadjuvant chemotherapy regime, degree of tumor differentiation, LDH, and CA19-9 were not significantly associated with survival. Our results also showed that ECOG performance status, distant metastasis, and NLR were independent predictors of PFS and OS among CRC patients receiving neoadjuvant CRT.

Several studies have been concluded that high NLR and low LMR are associated with worse survival in several kinds of malignancies^16,18,21–23^. This research confirmed the prognostic value of NLR in our study population. As previous studies reported, a prognostic factor with RR > 2 is considered to be useful, which indicated that NLR was a powerful predictive factor for CRC patients undergoing neoadjuvant CRT. This study also confirmed that LMR was significantly associated with PFS and OS in the univariate analysis, however, HR value showed no statistical significance about the tendency that elevated LMR was associated with favorable outcome in the multivariate analysis. Although precise mechanism is not completely clarified, several studies have suggested that neutrophils promote remodeling of the tumor microenvironment via production of cytokines and chemokines, which exert effects in tumor cell proliferation and metastasis^24–26^. Tumor-infiltrating lymphocytes play an important role in cancer immune-surveillance and cytotoxic cell death, and therefore suppress the progression of tumor^26–28^. Tumor-associated macrophages derived from circulating monocytes are reported to suppress adaptive immunity and therefore promote tumor growth and metastasis^29–31^. In addition, serum monocytes level could reflect the formation of tumor-associated macrophages in tumor microenvironment^32^. Thus, high neutrophils, high monocytes, and low lymphocytes are associated with poor outcome. In this way, both low NLR and high LMR ratio reveal favorable outcome.

Apart from hemostasis and thrombosis, platelets have reported to be responsible for tumor cell growth and metastasis by releasing platelet-derived growth factors and numerous pro-angiogenic proteins including vascular endothelial growth factor and proteases^33,34^. Reciprocally, tumor cells could induce the aggregation of platelet and manipulate platelet activity to facilitate tumor progression^33,35^. As a result, high serum platelets contribute to a poor outcome, in this way, high PLR which indicates high platelet counts and low lymphocyte counts is related to adverse prognosis. Several previous studies have reported the efficacy of PLR as a prognostic factor in CRC while several studies have reported that PLR is not an independent prognostic factor^13,19,20,36–38^. In the present study, results of univariate analysis revealed that pretreatment PLR was an index associated with PFS and OS, while results of multivariate analysis suggested that PLR was not an independent prognostic factor of survival. However, our results showed a tendency of improved survival among patients with low PLR than those with high PLR. Thus, the prognostic value of PLR is still controversial and therefore should be further interpreted.

SII was only recently investigated as a prognostic factor in several types of tumors, and its prognostic value in CRC patients has not been well defined so far^39–43^. Elevated SII indicates high neutrophils, high platelets and low lymphocytes. As mentioned above, high value of SII reflects both progression of cancer and weak immune status of host. In this study, results of univariate analysis proved that high SII was associated with poor outcome, although results of multivariate analysis suggested a tendency of improved PFS and OS in patients with high LMR which shown no statistical significance. Therefore, further studies are expected to confirm the prognostic value of SII.

This research had several limitations. Firstly, our study is a retrospective study, but complete data and regular follow-up can partly compensate for this limitation. Secondly, we obtained the hematological data of each patient within 4 weeks prior to receiving neoadjuvant CRT; however, value of inflammatory indexes may vary over time. Lastly, single-center study with a limited number of patients (n = 98) may cause selection bias, thus multi-center and larger population studies are needed to validate these results.

In conclusion, this research revealed that ECOG performance status, distant metastasis, NLR, PLR, LMR and SII were significantly associated with PFS and OS in CRC patients receiving neoadjuvant CRT. Furthermore, ECOG performance status, distant metastasis, and pretreatment NLR were shown to have independent prognostic value. We believed that pretreatment inflammatory indexes, especially NLR, could be good parameters for predicting survival of CRC patients receiving neoadjuvant CRT. However, further investigations are required to validate these results.

## Materials and Methods

### Patients and blood count parameters

We retrospectively reviewed a database of 7207 patients with CRC who were treated in the department of colorectal surgery at the West China Hospital from January 2010 to December 2015. The inclusion criteria included: (a) patients with CRC confirmed by histopathology, (b) patients who received neoadjuvant CRT, and (c) patients with available and complete clinical records including demographic data, pathologic characteristics of the tumor, laboratory data and therapeutic interventions. The following exclusion criteria were applied: (a) patients with clinical evidence of acute infection, chronic infection, systemic inflammation or other autoimmune diseases, (b) patients prior received immunosuppressive therapy or anti-inflammatory drug, (c) patients suffered from hematologic diseases, and (d) patients diagnosed with malignant disease primarily arising from other organs.

The data was extracted from patients’ medical records. Laboratory index value such as neutrophil counts, lymphocyte counts, platelet counts, monocyte counts, LDH, CA 19-9 and carbohydrate antigen-125 (CA-125) were obtained for each patient within 4 weeks prior to receiving neoadjuvant CRT. Patients were staged according to the tumor-node-metastasis (TNM) classification system of American Joint Committee on Cancer. NLR and PLR were defined as the ratio of absolute neutrophil counts and platelet counts divided by the absolute lymphocyte counts, respectively. LMR was calculated as the absolute counts of lymphocyte divided by the absolute monocyte counts. SII was defined as neutrophil counts × platelet counts/lymphocyte counts.

All patients were regularly followed as follows, every month in the first year, every 3 months in the second year and every 6 months thereafter. Follow-up started from the date of primarily receiving neoadjuvant CRT to March 2017 or the date of death. The primary endpoint was PFS and the secondary endpoint was OS. This study was approved by the Ethics Administration Office of West China Hospital, Sichuan University, and this research was performed in accordance with the relevant guidelines and regulations. An exemption from informed consent was also approved by this Ethics Administration Office.

### Statistical analysis

PFS was measured as the interval between the date of patients primarily receiving neoadjuvant CRT and the date when radiological evidence of recurrence was observed. OS was defined as the duration from the date of primarily receiving neoadjuvant CRT to the time of death from any causes or the date of last follow-up. Patients were divided into groups based on the median index value of NLR, PLR, LMR, and SII, respectively. We examined the differences of characteristics between groups by using the Student’s t-test for continuous variables; categorical variables were compared using the χ2 test or the Fisher exact test. Log-rank test was performed to identify the associations between inflammatory indexes and survival. Kaplan–Meier analysis was used to compare the survival curves between groups. Variables that were found to be significantly associated with survival by univariate analysis were further evaluated by using the Cox proportional hazard regression analysis. All p-values were based on two-sided testing and a p < 0.05 was considered to be statistically significant. Statistical analysis was performed by using SPSS version 21.0 (IBM Corporation, Armonk, NY, USA).

### Ethical approval

This study was approved by the Ethics Administration Office of West China Hospital, Sichuan University.
